# Recent Advances and Developments in Bacterial Endophyte Identification and Application: A 20-Year Landscape Review

**DOI:** 10.3390/plants14162506

**Published:** 2025-08-12

**Authors:** Neo M. Mametja, Thanyani E. Ramadwa, Muthumuni Managa, Tracy M. Masebe

**Affiliations:** 1 Department of Life and Consumer Science, College of Agriculture and Environmental Sciences, University of South Africa, Florida Campus, Johannesburg 1710, South Africa; ramaate@unisa.ac.za; 2 Institute for Nanotechnology and Water Sustainability (iNanoWS), College of Science, Engineering and Technology, University of South Africa, Florida Campus, Johannesburg 1710, South Africa; managme@unisa.ac.za

**Keywords:** culture-dependent techniques, culture-independent techniques, endophyte–host interactions, plant growth-promoting bacteria (PGPB), multi-omics approaches, sustainable agriculture

## Abstract

Bacterial endophytes have emerged as critical components of plant microbiomes, offering multifaceted benefits ranging from growth promotion to stress resilience. This review synthesizes two decades of research, from 2004 to 2024, on bacterial endophyte identification and applications, highlighting advances in both traditional culture-based techniques and modern omics approaches. The review also focuses on interactions between these microorganisms and their host plants, emphasizing their roles in biocontrol, phytoremediation, and nanoparticle biosynthesis. While significant progress has been made in characterizing cultivable bacterial endophytes, challenges persist in accessing unculturable species and understanding strain-specific functional mechanisms. The integration of metagenomics, metatranscriptomics, and metabolomics has begun unraveling this hidden diversity, revealing novel metabolic pathways and plant–microbe communication systems. There have been limitations in endophyte isolation protocols and field applications, and therefore a need exists for standardized frameworks to bridge lab-based discoveries with agricultural practices. Cutting-edge multi-omics techniques, such as genomics, transcriptomics, metabolomics, proteomics, and phenomics, should be used more in future research to clarify the mechanistic underpinnings of plant–endophyte interactions to thoroughly profile the microbial communities and unlock their functional potential under diverse environmental conditions. Overall, bacterial endophytes present viable paths toward sustainable farming methods, supporting food security and crop resilience in the face of environmental difficulties by providing a transformative opportunity for next-generation agriculture, mitigating climate-related agricultural stressors, reducing dependence on synthetic agrochemicals, and enhancing crop productivity.

## 1. Introduction

In different ways, diverse microorganisms (bacteria, fungi, archaea, and oomycota) are naturally connected to plants. Endophytic microorganisms are widespread in plants, and their relationships with host plants have been described as a balanced symbiotic continuum that ranges from mutualism to commensalism but not parasitism [1,2]. They have been reported to be found in nearly all parts of plants from all groups—algae, angiosperms, bryophytes, gymnosperms, lichens, and pteridophytes—including seeds in tall fescue (*Festuca arundinacea*) or other grasses, which are vertically transferred to the next generation [3]. Bacterial endophytes are one class that colonizes the internal tissues of the host plant, the stem, leaves, flowers, and fruits, as well as the seeds, thereby developing their lifestyle without causing harm to the host cells and showing no external signs of infection or negative effect on their host [4]. Bacterial endophytes benefit from plants in that plants provide a protected living space and a constant supply of nutrients [5,6]. In turn, bacterial endophytes form a beneficial relationship with their host plants, providing them with various benefits such as enhanced plant growth [7], improved phytoremediation efficiency [8], the conferring of host plant tolerance to abiotic and biotic stresses [9], nutrient uptake, hormone production, and protection against pathogens [10,11]. Mundt and Hinkel documented the first isolation of endophytic bacteria from the ovules and seeds of 27 plants [12]. Bacterial endophytes are commonly classified into two major divisions based on the plant-inhabiting life strategies they exhibit, viz., facultative and obligate endophytes [13]. Facultative bacterial endophytes are a group that have a stage in their life cycle where they dwell outside the host plant but may enter the host tissue, through infection or at the slightest opportunity, and alternate between host plant and the soil, while obligate bacterial endophytes are strictly hooked and live inside their host plant’s tissues for their growth [14,15]. The novelty of the present review lies in its exploration of bacterial endophyte dynamics and the status of current understanding pertaining to the recent advances and developments in bacterial endophyte identification. This study also explores traditional techniques, emerging techniques, and omic approaches, further highlighting the challenges and limitations in their identification.

## 2. Diversity and Distribution of Bacterial Endophytes

Bacterial endophytes can be isolated from two different regions: the part above ground of the plant (phyllosphere) and the part below ground (rhizosphere), characterizing each environment as harboring different bacterial communities shaped by the traits of each section. Diversity for the part below ground can be regulated by different factors associated with soil microbial communities and host factors. The microbiome composition within the root interior is significantly less diverse than that of the microbiomes in the soil [16]. Numerous studies have documented great bacterial endophyte diversity and further demonstrated that microbial communities are still far more diverse in the endosphere than they are in the rhizosphere [17,18]. The diversity of bacterial endophytes demonstrates that they relate to different plant species and have been found in every plant species studied. Thus, an endophyte-free plant is a rare exception in the natural environment. In fact, a plant without associated beneficial bacteria would be less fit to deal with phytopathogens and more susceptible to stress conditions [19,20]. A significant number of studies have analyzed and reported on the effects of different environmental variables on bacterial endophyte diversity and have shown that different plant hosts may harbor a similar population of bacterial endophytes [12]. Bacterial endophyte populations vary within the same species, from plant to plant, from species to species, and from region to region, and they differ with climatic conditions within the same region. Bacterial endophytes are important, and their distribution and population structure within host plants are considerably affected by a wide range of factors, such as the hosts’ genetic backgrounds, ages, and interactions between the inhabiting microorganisms, as well as the microbe–plant interaction and environmental conditions [21]. Plant compartments, genotypes, and geographic locations are vital factors that shape the endophytic microbiome composition; therefore, such environmental conditions, including availability of nutrients, pH, temperature, and humidity, which determine the distribution ranges of hosts, also determine the species distribution among the endophytes [22]. Studies on *Arabidopsis*, *Oryza*, *Populus*, and *Zea* validated that bacterial endophyte communities are generally influenced by a combination of the host environment and season, host genotype variation, and host species identity [23,24].

Additionally, single host plant species may accommodate multiple bacterial endophytic genera and species and the range of bacterial endophytic community depends widely on the colonized tissue, plant type, or endophyte isolation season. The technique (type, concentration, and even length of the treatment time with sterilizing agent) employed to investigate these bacterial endophytes is a significant determinant utilized to detect the host plant’s bacterial endophytic diversity [25]. Bacterial endophytes are often associated with a variety of plant organs, of which many may harbor similar species, depending on the diversity and composition of microbial communities. The most described genera in the literature of bacterial endophytes, and also common inhabitants of the rhizosphere, are *Azospirillum*, *Bacillus*, *Burkholderia*, *Enterobacter*, *Microbacterium*, *Micrococcus*, *Pantoea*, *Pseudomonas*, *Stenotrophomonas*, and *Serratia*; thus suggesting that endophytic microbiome could be a subpopulation of the rhizosphere-inhabiting bacteria [26,27,28,29,30,31].

## 3. Biological Roles, Benefits, and Prospective Application of Bacterial Endophytes

**Figure 1:**
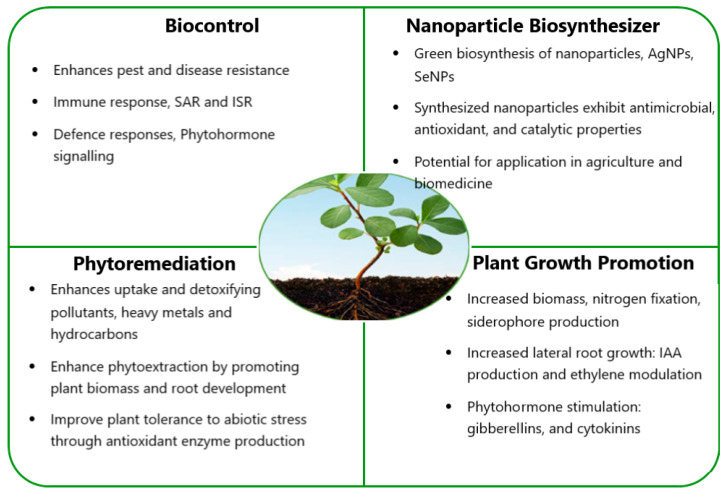
Overview of beneficial roles and applications of bacterial endophytes.

Bacterial endophytes use direct or indirect techniques to benefit their host plants in various ways. The ability of bacterial endophytes to produce bioactive compounds of agricultural, industrial, and pharmaceutical importance makes them interesting research candidates. They play important roles in different fields of life, including impacting host plants and affecting the environment and human life, thus encompassing a range of beneficial applications in agriculture, biotechnology, and environmental management, as shown in Table 1 and Figure 1 [9,32]. The crosstalk between key mechanisms used by bacterial endophytes to benefit the host plant largely contributes to and accounts for signal transduction. Mechanisms that bacterial endophytes employ for plant growth and development comprise processes that include biocontrol [33], bioremediation [34], biotic/abiotic stress reduction [35], endosphere colonization [36], enzyme secretion [37], and the induction of plant-resistance [38]. The production of antibiotics, cell wall-degrading enzymes, decreased plant ethylene levels, reduced iron availability to pathogens, and the synthesis of pathogen-inhibiting volatile compounds are a few examples of the numerous common mechanisms used by bacterial endophytes to promote plant growth and development [39]. Furthermore, bacterial endophytes can indirectly enhance plant growth by stimulating plant responses or producing secondary metabolites that act against phytopathogens [40].

### 3.1. Biocontrol Role

Biocontrol of plant pathogens (phytopathogens) has gained significant attention in recent years due to its potential as a sustainable and environmentally friendly alternative to chemical pesticides, and one such approach involves the use of bacterial endophytes, which are beneficial bacteria that reside within plant tissues. The design and development of novel antimicrobial agents have attracted considerable attention due to adverse impacts associated with conventional agrochemical pesticides (fungicides, insecticides, herbicides, and nematicides), such as phytotoxicity, pathogen resistance, and chemical residues exposed to host plants [66]. Agrochemicals execute effective roles to ensure a continuous dynamic agricultural structure and control of plant diseases, but extensive use of agrochemicals results in adverse outcomes for the functioning of the environment, agricultural prosperity, and humans. Bacterial endophytes possess several mechanisms that secrete biocontrol agents to inhibit entry and control the growth of phytopathogens, among other activities, such as competition for nutrients, production of antimicrobial compounds, and induction of plant defense responses [67,68]. Furthermore, other studies have presented data on the impact of bioinoculant and endophyte application on development and growth for nutrient absorption in corn, cotton, sorghum, and tomato [69,70]. Certain bacterial endophytes, such as *B. amyloliquefaciens*, *B. pumilus*, *B. subtilis*, *Pseudomonas fluorescens*, *P. syringae*, and *Serratia marcescens*, assist their host plants to elicit antibiotics and induce systemic resistance (ISR), resistance mechanisms which occur when plants successfully activate their defense mechanisms in response to infection by a phytopathogen [71,72]. *Bacillus* spp. demonstrated antagonistic activity against *Xylella fastidiosa*, which causes olive quick decline syndrome (OQDS) in olive trees [73,74]. Moreover, novel *Streptomyces* spp. MBFA-172 in strawberry plants was reported to exhibit biocontrol activity against *Glomerella cingulata* [75]. *Bacillus amyloliquefaciens*, *B. subtilis*, *B. velezensis*, *Lactococcus lactis*, and *Leuconostoc mesenteroides* are some well-documented seed-associated bacterial endophytes to be used to treat bacterial wilt of tomato that exhibit biocontrol agent properties [76]. Studies by other teams also reported the ability of *B. velezensis* to inhibit the growth of a variety of fungal phytopathogens, such as *Colletotrichum coccodes*, *Fusarium avenaceum*, *Phoma foveat*, and *Rhizoctonia solani*, in potato in both in vitro and field experiments [77,78]. Therefore, isolates of bacterial endophytes can commercially be incorporated to synthesize biopesticides to both protect host plants and maintain a healthy environment [79]. Another study demonstrated that *B. velezensis* IALR619 has the potential to inhibit strawberry pathogen growth in a greenhouse and possibly increase fruit yield in the field, which confirmed that *B. velezensis* is a typical biocontrol agent used to control various soil-borne diseases as well as a plant growth-promoting bacteria [80,81]. Furthermore, some biocontrol agents possess plant growth-promoting capabilities, and their use can help reduce reliance on synthetic chemicals, minimizing environmental damage and promoting sustainable agricultural practices. As research in this field continues to progress, exploiting the potential of bacterial endophytes for biocontrol purposes holds great promise for the future of agriculture.

### 3.2. Nanoparticle Biosynthesizer Role

Biosynthesis and the production of nanoparticles (NPs) using bacterial endophytes have emerged as cutting-edge technologies due to the various functions, environmental clean-up, potential bioactivity, non-pathogenic nature, and enormous therapeutic applicability of these particles [82]. Biological approaches of NPs synthesis in place of physical and chemical approaches have been reported to have numerous advantages; they are easy, economical, safe, and eco-friendly because they require less energy and produce no harmful waste [83]. Biocompatible and non-toxic NPs are preferred when it comes to drug delivery and medicine. Therefore, their synthesis could easily utilize bacterial endophytes, and reports have revealed that numerous researchers have employed prokaryotic and eukaryotic endophytes for the synthesis of gold, silver, copper metal, and metal oxide NPs, which have been used in all domains of science [84]. Bacterial endophytes reduce metallic ions during biosynthesis and produce NPs. Furthermore, other researchers have reported NPs’ synthesis with other elements such as zinc sulfide, copper oxide, cobalt oxide, and nickel oxide from endophytes that were isolated from both terrestrial and marine environments. There are numerous potential uses for these biosynthesized NPs in nanomedicine because of their antibacterial, antifungal, antioxidant, antimicrobial, antidiabetic, anticancer, and photocatalytic degradation properties [85,86]. AgNPs synthesized using endophytic *Streptomyces* spp. showed antimicrobial, antioxidant, and larvicidal activities [87]. Low concentrations of AgNPs synthesized by *Pantoea ananatis* showed antimicrobial activity against *Candida albicans* and *Bacillus cereus*, and higher concentrations were active against multidrug-resistant strains of *Enterococcus faecium*, *Escherichia coli*, *Streptococcus pneumoniae*, and *Staphylococcus aureus* [88,89]. The endophytic *Streptomyces* spp., isolated from medicinal plants, synthesized CuNPs and CuONPs with antibacterial, antifungal, antioxidant, and insecticidal properties [90,91]. Fadiji and their team demonstrated the function of various NPs synthesized from bacterial endophytes in sustainable agriculture by enhancing plant growth and development, thereby strengthening resistance to disease [92]. Other studies documented that bacterial endophytes may serve as bio-factories for NPs, and those synthesized from them have immense potential in healthcare applications [93]. Silver NPs are widely used, specifically in bio-labeling, antimicrobial agents, catalysts, and sensors due to their unique optical, electrical, and magnetic properties [94].

### 3.3. Plant Growth-Promotion Role

One key area of interest in the study of bacterial endophytes is their role in promoting plant growth through enhancing disease resistance, nutrient uptake, and stress tolerance, which are attributed to several mechanisms employed by endophytes, such as the production of phytohormones (auxin, cytokinin, ethylene, and gibberellin), siderophores, and various enzymes that aid in nutrient acquisition, including nitrogen fixation, phosphate solubilization, and iron and potassium mobilization [95,96]. Additionally, bacterial endophytes can also facilitate plant growth by inducing systemic resistance against phytopathogens and pests by secondary metabolite synthesis and antibiosis, ultimately boosting plant responses to environmental stressors, leading to healthier host plants and surrounding environments [97,98]. Bacterial endophyte *Pseudomonas* spp. has been shown to stimulate pea plant growth and exhibits plant-beneficial traits such as phosphate solubilization and the production of indole-3-acetic acid (IAA), siderophores, hydrogen cyanide (HCN), and ammonia (NH_3_) [99]. Certain bacterial endophytes genera, including *Acinetobacter*, *Bacillus*, *Micrococcus*, *Paenibacillus*, *Pseudomonas*, *Pantoea*, and *Staphylococcus*, have demonstrated the ability to thaw dormant seeds, enhance seedling growth, protect against phytopathogens, and promote plant development [100,101]. These beneficial properties may explain why such microbes are consistently transmitted to subsequent plant generations through seeds [102]. Bacterial endophytes also play a significant role in protecting plants from pathogens, including bacteria, fungi, and nematodes [103]. Notably, the protein BphKLB400 from *Burkholderia xenovorans* LB400 confers herbicide tolerance onto pea plants and facilitates the detoxification of multiple herbicides [104]. Under abiotic stress conditions such as drought, salinity, and heavy metal exposure, enzymes like 1-aminocyclopropane-1-carboxylate (ACC) deaminase and IAA, along with their phosphate-solubilizing traits, help plants adapt and maintain their growth [105]. IAA contributes directly to plant physiological processes like chlorophyll content, increased biomass, and root development, while ACC deaminase modulates ethylene levels and mitigates growth inhibition under stress. Endophytes also promote drought tolerance by producing abscisic acid (ABA), volatile compounds, and osmoprotectants, improving plant water retention and osmotic adjustment [106]. Taxonomically, bacterial endophytes are diverse, encompassing Gram-positive and Gram-negative genera such as *Achromobacter*, *Agrobacterium*, *Bacillus*, *Pantoea*, *Xanthomonas*, and others. Many of these microbes produce bioactive compounds with antimicrobial and anticancer activities, positioning them as valuable resources to treat a range of illnesses [107,108]. Moreover, their bioactive metabolites have been reported to exhibit antidiabetic, antifungal, immunosuppressive, and anti-inflammatory properties [109,110,111,112]. The production of ACC deaminase is one of the key attributes of bacterial endophytes bacteria that stimulates plant growth under high concentrations of toxic metals, and lowers ethylene levels during stress, while simultaneously facilitating mineral solubilization [113,114]. Other findings have also highlighted endophyte-triggered defense responses; *Arthrobacter agilis* produces N,N-dimethyl hexadecylamine, a compound that induces iron uptake by roots against the growth of pathogenic fungi and defense-related gene expression in *Medicago truncatula*, offering protection against *Botrytis cinerea* and *Pseudomonas syringae* without involving the jasmonic acid (JA) pathway [115,116]. Additionally, ACC deaminase and IAA interaction are critical in modulating ethylene levels. Excess ethylene stimulated by IAA-induced ACC synthase activity can inhibit plant growth, which is mitigated by microbial ACC deaminase [117]. The application of the plant growth-promoting endophytes producing this enzyme has been linked to enhanced tolerance to various environmental stresses [118]. Specific endophytic strains such as *Pantoea agglomerans* aid in salt stress tolerance through IAA production, while *Bacillus licheniformis* promotes overall plant health, inhibits fungal parasites, and synthesizes gibberellins [119,120]. Similarly, *B. stratosphericus* has been demonstrated to suppress bacterial and fungal parasites and aid plants in the phytoremediation of heavy metals and other pollutants, including salt [34,121]. Thus, understanding these diverse mechanisms through which bacterial endophytes promote plant growth lays the foundation for the application of bacterial endophytes in environmental biotechnology and sustainable agriculture.

### 3.4. Phytoremediation Role

Phytoremediation is a green and sustainable technology that employs plants and their associated microorganisms to remove hazardous organic and inorganic pollutants from the environment [122,123]. Plants have the inherent ability to degrade certain pollutants, and phytoremediation using plants has been recognized as a cost-effective and eco-friendly approach for remediating contaminated sites. The success outcome of phytoremediation largely depends on the combined ability of plants and their bacterial endophytes to tolerate high levels of contamination [124,125]. The microbial degradation of inorganic compounds can result in the production of environmental metabolites such as carbon dioxide, humus, salts, water, and other beneficial by-products. In addition, bacterial endophytes have been shown to degrade environmental pollutants such as explosives, herbicides, and hydrocarbons [126,127]. In plant–endophyte interactions, plants offer nutrients and shelter to bacterial endophytes. In turn, bacterial endophytes with appropriate metabolic activity and degradation pathways enhance the breakdown of organic pollutants while reducing phytotoxicity and evapotranspiration. Moreover, plant growth-promoting endophytes further improve plant development and stress adaptation, especially in contaminated soils and water bodies [128].

Many bacterial endophytes are known for their ability to degrade xenobiotic pollutants, including alkanes, hydrocarbons, and pesticides, while promoting plant growth through mechanisms such as auxin production, nitrogen fixation, and siderophore secretion [129]. Some bacterial endophytes produce IAA and other bioactive compounds that support plant growth and facilitate remediation in heavy metal-contaminated environments [130,131]. Notably, phosphate-solubilizing bacterial genera such as *Pseudomonas*, *Burkholderia*, *Paraburkholderia*, *Novosphingobium*, and *Ochrobactrum* have demonstrated the ability to enhance biomass yield in certain seedlings due to their multifunctional traits. The development of bioinoculants using root-associated endophytes shows great promise in modern agriculture [132]. Given the extensive research on plant growth-promoting bacteria, their integration into organic farming systems has the potential to significantly boost agricultural productivity and contribute to global food security. Experimental bioremediation studies have revealed that the bacterial endophyte *Paenibacillus* spp., isolated from *Tridax procumbens*, achieved significant heavy metal removal, eliminating up to 59.4% of copper and 51.4% of zinc, demonstrating its potential for multi-metal remediation [51,133,134]. Further research is needed to identify novel bacterial endophytes with strong contaminant-degrading abilities. Optimizing their potential can enhance sustainable plant health and contribute to environmentally friendly agricultural practices aimed at improving crop productivity and ecosystem resilience.

## 4. Isolation Techniques

Several researchers have extensively studied and reviewed various techniques for isolating bacterial endophytes from various plant tissues, such as seeds, roots, stems, leaves, and flowers [135,136]. The most widely accepted isolation technique is whole-surface sterilization, which involves three main steps: (1) surface sterilization of plant tissues, (2) maceration of the sterilized tissues, and (3) culturing of serially diluted macerates [16,137,138]. Surface sterilization is a critical first step of isolation that eliminates all potential pollutants, epiphytic microorganisms, external contaminants, and other debris. This is typically achieved by sequential treatment of the plant tissues with sterilizing agents, such as ethanol, hydrogen peroxide, sodium or mercuric chloride, and hypochlorite solutions, for a predetermined period to improve the effectiveness of the sterilization procedure, followed by thorough rinsing with sterile distilled water to remove any residual chemicals [139,140]. The effectiveness of sterilization is verified by plating the final rinse water on a culture medium, where the absence of bacterial growth confirms the effectiveness of the sterilization procedure and any observed growth denotes the availability of unwanted pollutants [125,141]. A high yield of bacterial colonies after sterilization indicates minimal impact on the internal endophytic population [142,143]. An ideal sterilization protocol must balance the complete removal of surface microbes while preserving the viability of internal endophytes. The sterilizing agents ought to eliminate the epiphytic microbes without causing any damage to the endophytes or host tissue, though this balance is often difficult to achieve because aggressive sterilizing agents can sometimes penetrate plant tissues and harm the endophytes.

Surface sterilization methods may vary depending on the plant species, tissue type, and researcher’s preference; however, the most commonly used surface-sterilization protocols followed are those outlined by [144,145,146]. Mashiane et al. demonstrated the surface sterilization of explants using a three-step approach that involved immersion in 70% ethanol for 60 s, followed by rinsing with distilled water and subsequent sterilization in 3% sodium hypochlorite (NaClO) for 60 s, and finally in 70% ethanol for 30 s. Samples were further washed in sterile distilled water three times, for 60 s each time. The process involved the inoculation of explants obtained from different parts of the maize plants on nutrient agar, tryptone soy agar, and nutrient broth, and incubated at 27 °C for 24 h to isolate the bacterial endophytes [147]. In another study, Dasgupta et al. sterilized pooled leaf samples from Eucalyptus clones with 70% ethanol (10 min), 2% NaClO (15 min), and 70% ethanol (10 min), followed by eight sterile water washes. The sterilized leaves were plated on Luria–Bertani (LB) agar to confirm sterility and later used for genomic DNA extraction [148]. Similarly, Abbamondi et al. reported on the isolation of 12 bacterial strains from fresh roots using a protocol involving washing with P-buffer containing Tween 40, sterilization in 5% NaClO (5 min), 5 sterile water rinses, tissue maceration in 10 mM MgSO_4_, and plating serial dilutions on LB medium. Surface sterility was confirmed by plating the final rinse water [149].

## 5. Identification and Characterization of Bacterial Endophytes

Accurate identification is essential to understand the diversity, functionality, and potential applications of bacterial endophyte populations in plants. Numerous studies have well-documented diverse approaches for the assessment of these microorganisms, and a summary of some techniques used to study bacterial endophytes is presented in Table 2. Research in this area generally employs both culture-dependent and culture-independent techniques as cornerstone methods for identification and characterization. These typically involve (i) basic culturing, (ii) DNA extraction, (iii) PCR amplification using specific primers, (iv) sequencing, and (v) comparison of the obtained sequences with those available in public databases using NCBI BLAST+ v2.16.0 [150,151,152].

### 5.1. Culture-Dependent Techniques

Traditionally, culture-dependent techniques have been utilized to obtain pure bacterial isolates for studying the roles, characteristics, and functional traits of bacterial endophytes. These methods have contributed significantly to the identification and characterization of the diversity of plant-associated bacterial endophytes; however, a key limitation is that they capture only a small fraction of the total microbial population, leading to a biased representation of the actual diversity of endophytic populations [153,154]. Culturable bacterial endophytes are identified and characterized through biochemical, morphological, physiological, and molecular approaches [155,156]. These techniques are inherently restricted to microorganisms that can be cultivated under both in vitro and in planta conditions, excluding the vast majority of unculturable microbes. As a result, culture-dependent approaches do not provide a comprehensive overview of the bacterial endophyte population [157,158]. Some studies analyzed bacterial endophyte diversity in various tissues of *Dendrobium officinale* and demonstrated that culture-dependent methods yielded a skewed and limited diversity profile. Comparable observations have been reported in other studies, which found that these methods tend to underestimate microbial diversity when compared with culture-independent approaches [153,159].

Bacterial colonies exhibiting distinct morphologies are typically sub-cultured on freshly prepared nutrient media and preserved on nutrient agar slants for downstream analyses. The composition of the nutrient medium plays a critical role in supporting bacterial growth, with carbon- and nitrogen-rich media such as nutrient agar and potato dextrose agar commonly used to encourage the proliferation of bacterial endophytes [160]. In addition to culturing, microscopy and staining techniques can be employed to visualize the colonization patterns, morphology, and cellular structures of bacterial endophytes. Various types of microscopes, including transmission electron microscopes and light microscopes (bright-field, fluorescence, and laser scanning microscopes), have been instrumental in bacterial endophyte identification [158,161]. These techniques are frequently complemented by biochemical assays to evaluate the metabolic and physiological capabilities of bacterial endophytes. Despite their value, culture-dependent approaches have significant limitations. They only allow the recovery and analysis of culturable microorganisms, leaving most unculturable endophytic populations unexplored. It is estimated that only around 5% of bacterial endophytes have been identified using conventional culturing techniques, as many bacterial strains have highly specific metabolic and physiological requirements that are challenging to replicate in vitro [162,163].

### 5.2. Culture-Independent Techniques

The literature has supported the occurrence of bacterial endophytes in host plants through various culture-independent assays [164]. Researchers can examine the diversity and functionality of bacterial endophytes using a vast array of molecular techniques once they have been isolated. Culture-independent techniques bypass the need for cultivation, providing a fast and accurate taxonomic landscape of bacterial endophytes. This enables the study of microbial communities in their natural environments [160]. As high-throughput techniques, they offer a more comprehensive understanding of endophyte diversity and community structure. Additionally, culture-independent techniques have elucidated the makeup of bacterial communities in a variety of plants, including bananas, beans, rice, and medicinal plants such as aloe vera [112,165]. Molecular and visualization approaches, as examples of culture-independent techniques, enable the identification of unculturable bacterial endophytes within host plants, including those that grow slowly or are present in low abundance and are thus overlooked by culture-dependent methods [166,167,168]. Microscopy is the most direct method for observing bacterial endophytes, and the exact location of these endophytes within plant tissue can be determined using both electron and light microscopy [169,170]. While light microscopy provides a general overview of the structures and morphology, it is limited in resolution compared to electron microscopy, which enables high-resolution ultrastructural analysis. Electron microscopy helps detect bacterial endophytes, reveal their extent of colonization, and visualize bacteria–host interactions and their establishment within the plant environment. Scanning electron microscopy (SEM) and transmission electron microscopy (TEM) yield valuable information about the surface and internal structures of bacterial endophytes, respectively [155,171,172].

Morare et al. morphologically identified bacterial endophytes from *Crinum macowanii* using Gram-staining, and high-resolution images were created using SEM micrographs to detail the shapes and confirm identities. Macroscopic identification of bacterial endophyte colonies involved assessing colony color, consistency, elevation, margin, opacity, and surface structures. Isolates were further subjected to a Gram-stain reaction and viewed under an oil immersion compound bright-field microscope at 100× magnification [173,174]. Biochemical assays such as catalase, oxidase, urease, coagulase, and starch hydrolysis tests are also employed to validate the biochemical activity of bacterial endophytes [3,164]. However, the use of different culture-independent techniques depends on the experimental design and research objectives. The choice of one technique over another is often subjective, based on the researcher’s hypothesis and available resources. A thorough understanding of the advantages and limitations of each technique increases the possibility of obtaining comprehensive data and insights. Advancements in meta-omics provide modern tools to better understand the activities of bacterial endophytes in plants [175,176]. Ultimately, the most effective approach might involve combining modern techniques with traditional methods, a strategy that has been shown to reduce error rates in some next-generation sequencing methods and improve data quality.

#### Meta-Omics Approaches

- *Metagenomics*

Metagenomics utilizes modern genomic techniques to analyze genome sequences obtained directly from various environments, providing insights into the diversity, genetics, and physiology of unculturable microorganisms. This approach has been a widely used method for the study of microbiome populations, as it examines both single marker genes and the complete genomes of all organisms present, providing valuable information on genetic structure, novel genes, functional roles, and evolutionary relationships within microbial communities [177,178]. The application of metagenomic techniques has demonstrated significant potential in identifying the role of bacterial endophytes in essential plant biological processes, such as biodegradation, nitrification, plant growth promotion, phosphate solubilization, phytoremediation, and phytopathogens suppression. Compared to traditional culture-dependent methods, metagenomics provides an unbiased perspective, although it comes with limitations, such as high sequencing costs, interference from plant DNA, low extraction efficiency, and low DNA yields [179,180,181]. Beyond community composition, shotgun metagenomic sequencing provides deeper insights into the functional potential of endophytes. Tian et al. employed shotgun metagenomic sequencing alongside detailed taxonomic and functional profiling to compare bacterial endophyte communities in healthy tomato roots and roots infected with nematode *Meloidogyne incognita*. The study revealed that functional annotation of microbial genomes uncovered genes involved in the biosynthesis of secondary metabolites, including those with nematicidal, and plant-protective activities (e.g., IAA production, plant polysaccharide degradation, and nitrogen fixation). These findings suggest that the enriched endophyte populations in infected roots harbor enzymatic pathways and metabolites that can suppress nematode colonization. Thus, this pioneering study not only illuminates the dynamic shifts within the tomato root endosphere during nematode infection but also identifies microbial secondary metabolites as potential biological control agents against *M. incognita* [182]. Similarly, functional genes associated with nitrogen metabolism, IAA and tryptophan biosynthesis, siderophore production, and phosphate solubilization have been identified in root-associated endophytic microbiomes in maize using shotgun sequencing. High-throughput sequencing technologies, such as Illumina and 454 pyrosequencing have been utilized to explore diverse bacterial endophytes in oak and sorghum plants [182,183]. Furthermore, shotgun metagenomic sequencing, coupled with bioinformatics analysis, revealed taxonomic composition and predicted functional genes involved in plant growth promotion. In the *Panax ginseng* bacterial community, genes related to ACC deaminase activity, phosphate solubilization, 4-phytase, methanol utilization, and nitrogen metabolism have been identified using metagenomic analysis. Additionally, metagenomics has facilitated the study of genes responsible for bacterial attachment, carbohydrate metabolism, responses to temperature fluctuations, osmotic stress, pH regulation, and secretion systems [184,185,186].

b. *Metatranscriptomics*

Metatranscriptomics focuses on studying the expression of all messenger RNA (mRNA) transcripts in microbial cells associated with various plants. This technique provides insights into transcribed genes, enabling the understanding of genetic profiles and functional similarities or differences among bacterial endophytes in diverse environments [151,187,188]. RNA sequencing (RNA-seq) allows comprehensive transcriptome analysis of microbial communities under different conditions, establishing a direct link between the microbial genetic composition and the functional roles of expressed genes. These techniques have been widely used in plant–microbe interaction studies, as they characterize active microbial genes with specific functions and identify those driving symbiotic relationships between microbial communities and host plants. In this context, proteins play a key role in promoting plant growth under stress conditions by acting as precursors for various bacterial endophyte-induced mechanisms [189,190]. Moreover, stress-responsive genes from bacterial endophytes can act as catalytic regulators of microRNAs (miRNAs), which modulate the expression of essential genes involved in plant responses to abiotic stress in crops such as wheat, rice, Arabidopsis, and Medicago. miRNA169 and miRNA169c have been reported to enhance stress responses in tomato and rice plants. Additionally, some studies have also explored the role of miRNAs in mediating metal stress in two rice subspecies, *Indica* and *Japonica,* using RT-PCR analysis [191,192,193,194]. Bacterial endophytes also contribute to plant stress tolerance through the secretion of ACC deaminase, making them promising candidates for bioinoculants that support plant survival under challenging conditions such as drought, salinity, and extreme temperatures. The effectiveness of metatranscriptomic studies is enhanced when combined with metagenomic approaches. While metagenomics identifies genes present and absent in culturable and non-culturable bacteria, metatranscriptomics compares the transcriptomes of related bacterial species, shedding light on microbial community responses to environmental changes. Together, these techniques provide valuable insights into bacterial endophytes within ecosystems, potentially leading to the discovery of novel genes and their functions [187,195]. One key advantage of metatranscriptomics is that it does not require probes or primers, allowing for unbiased sequencing of microbial transcripts. Additionally, it provides information on the expression of non-coding genes and small RNAs, further enhancing its utility for microbial profiling. As direct sequencing methods advance, metatranscriptomics will continue to expand microbial community databases, helping to address emerging research questions. Despite its benefits, this method has several drawbacks, such as the inability to recover mRNA transcripts due to the instability and difficulties of metatranscriptomics. Additionally, the separation of mRNA from other RNA types, including tRNA, rRNA, and miRNA, remains a complex process. The presence of humic compounds (humic acids, fulvic acids, and polyphenols) that co-exist with nucleic acids can also interfere with accurate sequencing results [196,197,198,199].

c. *Metaproteomics*

Metaproteomics studies gene expression at the protein level within microbial communities, offering insights into their functional activities, including biochemical processes, bioremediation, and degradation pathways. This technique allows for the classification and quantification of proteins from bacterial endophytes, aiding in the understanding of gene functions, DNA transcription to mRNA, and subsequent protein translation [151,187,188]. Metaproteomics provides cutting-edge capabilities for analyzing microbial functions under biotic and abiotic conditions, such as enzyme activity in metabolic pathways, protein identification, and osmotic regulation. In one study, metaproteomic analysis identified the bacterial endophyte *Gluconacetobacter diazotrophicus* in drought-prone sugarcane soils. Another study compared the metaproteomic profiles of ratoon sugarcane in the rhizosphere with those of the plant itself, revealing that 24.77% of the proteins found in the soil originated from bacteria, with many upregulated proteins linked to membrane transport and signal transduction [150,200]. Metaproteomics has emerged as an essential tool for linking microbial taxonomic diversity with functional profiles. However, analyzing proteomes in mixed microbial communities remains challenging because of their complexity, with only a small fraction (approximately 1%) of the total metaproteome typically identified. The absence of genetic data further complicates microbial proteome studies, rendering them incomplete. Additionally, protein extraction and sample preparation are hindered by interfering substances such as alkaloids, organic acids, polysaccharides, polyphenols, lipids, and secondary metabolites. Despite these challenges, metaproteomic studies have provided valuable findings. Other teams identified diazotrophic methanotrophs in rice roots using metaproteomic analysis, while bacterial proteins linked to amino acid, carbohydrate, and lipid metabolism have been reported in endophytic genera such as *Actinobacteria*, *Bacteroidetes*, *Firmicutes*, and *Proteobacteria* [9,201,202]. Metaproteomics has been applied in environmental research, particularly in soil–microbe and plant–microbe interactions, highlighting its potential in agricultural biotechnology research. Analyzing the genomes of endophytes from plants in drought-prone soils could help predict specific functional protein genes associated with bacterial and fungal adaptations [188,200,203]. However, metaproteomics is still in its early stages, and the literature on the protein expression of bacterial endophytes in major crops remains limited. Future research should focus on expanding metagenomic data from diverse microbial environments to enable a more comprehensive characterization of bacterial endophyte communities. Advancements in metaproteomic techniques will be crucial for unveiling the protein functions and metabolic pathways of endophytic bacteria, ultimately enhancing their application in sustainable agriculture [204,205,206].

d. *Metabolomics*

Metabolomics focuses on the study of metabolites in living cells and has been widely applied across various biological fields, although there is less literature available documenting its use in identifying the functional characteristics of microbial endophytes [5,207]. Research has documented numerous bacterial metabolites analyzed using techniques such as capillary electrophoresis–mass spectrometry (CE-MS), liquid and gas chromatography–mass spectrometry (LC-MS and GC-MS), and nuclear magnetic resonance spectroscopy (NMR). In plants, metabolites are chemical compounds produced through metabolic pathways that play essential roles in growth, development, and defense mechanisms [186,208,209]. The application of metabolomics in crop production holds great potential for determining metabolite composition at different developmental stages. Bacterial endophytes contribute significantly to plant growth and survival under extreme environmental conditions by producing metabolites that support adaptation and survival in these conditions. Studies investigating the metabolic potential of bacterial endophytes have revealed diverse functional secondary metabolites that drive microbial adaptation, interactions, and lifestyle shifts in plant-associated environments [178,210,211]. Notable metabolites produced by bacterial endophytes include sespenine (*Streptomyces* spp.), spoxazomicins (*Streptosporangium oxazolinicum*), siderophores (*Pseudomonas aeruginosa*), serobactin A (*Herbaspirillum seropedicae*), valienamine (*Burkholderia kirkii*), pavettamine (*Burkholderia* spp.), and coronatine (*Pseudomonas syringae*). Microbial metabolite profiling plays a crucial role in identifying novel compounds involved in cellular processes and microbial interactions. This approach also offers potential for the discovery of alternative antibiotics derived from bacterial endophytes, contributing to advancements in biotechnology and medicine [202,212,213,214]. Table 3 depicts some of the omics tools and databases that have been documented for bacterial endophyte studies.

## 6. Literature Search Criteria

### 6.1. Review Strategy

To ensure a comprehensive and objective review process in accordance with the PRISMA 2020 guidelines, an extensive search of the literature on bacterial endophytes was conducted using multiple electronic databases, including PubMed, Scopus, Web of Science, ScienceDirect, Google Scholar, SpringerLink, and relevant master’s and doctoral theses were consulted [226]. The literature search used targeted keywords related to bacterial endophyte diversity, isolation techniques, molecular identification, and biotechnological applications.

### 6.2. Inclusion and Exclusion Criteria

**Figure 2:**
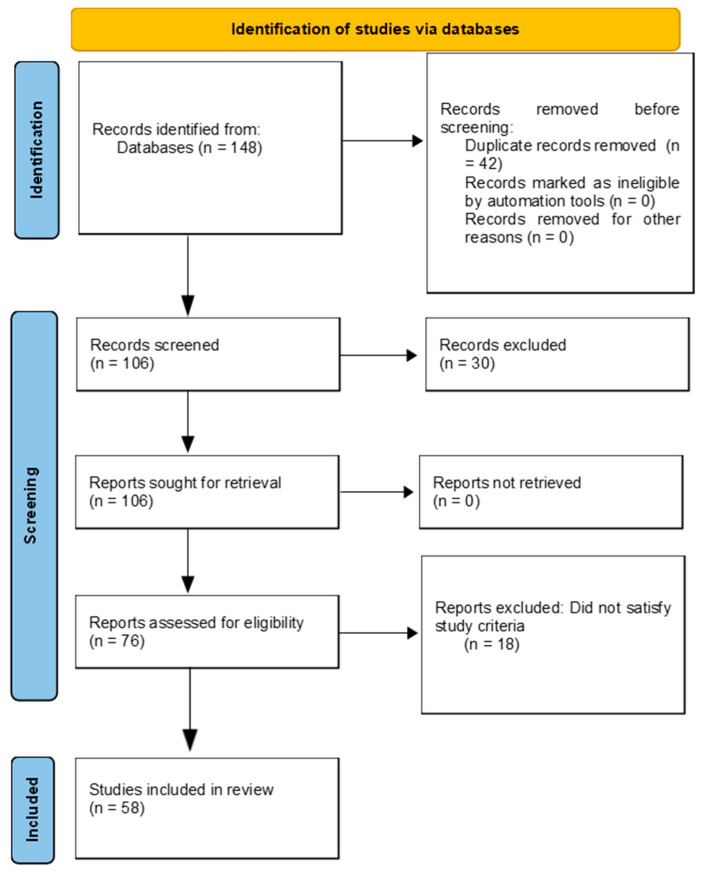
Survey flow diagram of the literature search and selection process.

To ensure relevance and quality, the following criteria were applied during literature screening. Studies included were those (i) published in English only; (ii) published between 2004 and 2024; (iii) that addressed bacterial endophyte diversity, isolation, identification, and application; and (iv) that involved plants that are agriculturally, ecologically, or medicinally significant. Exclusion criteria included studies focusing exclusively on fungal endophytes or rhizospheric microbes, non-peer-reviewed sources, non-English articles, and those without accessible full texts, as shown in Figure 2.

## 7. Conclusions and Future Outlook

Bacterial endophytes have emerged as pivotal components of plant-associated microbiomes, offering a wide array of benefits, including enhanced plant growth, improved tolerance to biotic and abiotic stresses, and promising applications in phytoremediation and biocontrol. Their potential to contribute to sustainable agriculture, reduce reliance on chemical use, and support environmental resilience is increasingly recognized. However, despite significant advances in characterizing their taxonomic diversity and functional roles, key knowledge gaps remain. The molecular mechanisms of plant–endophyte interactions are still not fully understood, particularly the signaling pathways, regulatory networks, and metabolic pathways that mediate the molecular crosstalk in beneficial symbiosis. Limitations in cultivation techniques, coupled with host-specific responses, and strain-level variability, pose challenges to their broader application. These complexities highlight the urgent need for innovative strategies that integrate both culture-dependent and culture-independent approaches.

Future research should prioritize elucidating the mechanistic basis of plant–endophyte interactions by utilizing advanced multi-omics approaches, such as genomics, transcriptomics, metabolomics, proteomics, and phenomics; to comprehensively profile the microbial communities and decode their functional potential under various environmental conditions. Such integrative approaches will not only facilitate the discovery of novel bioactive compounds and stress-responsive traits but also enable the development of precision bioinoculants tailored for specific crops and environments. To translate the scientific laboratory research into field applications, standardized protocols for microbial isolation, characterization, formulation, and application must be established. Furthermore, rigorous field trials will be needed to validate efficacy under diverse agroecological conditions, and the formulation of supportive regulatory and policy frameworks will also be essential to guide the safe and effective deployment of microbe-based technologies at scale. Ultimately, bridging the gap between foundational research and field-level implementation will critically require interdisciplinary collaboration between stakeholders. Exploration of untapped plant microbiomes, especially in extreme or understudied ecosystems, may yield novel strains with unique metabolic and adaptive traits, further expanding the functional resource base of microbial solutions for agricultural innovation.

In conclusion, bacterial endophytes represent a transformative opportunity for next-generation agriculture to mitigate climate-related agricultural stressors, reduce dependence on synthetic agrochemicals, enhance crop productivity, and secure global food systems. Their successful integration into modern agricultural systems offers a sustainable pathway toward food security and environmental resilience.

## Figures and Tables

**Table 1:** Beneficial association between bacterial endophytes and host plant.

Role of Beneficial Bacteria	Bacterial Endophyte	Host Plant	References
Enhance plant resistance to phytopathogens	*Bacillus amyloliquefaciens*	*Ginkgo biloba* and *Panax notoginseng*	[41,42]
	*Bacillus* sp.	*Curcuma longa*	[43]
	*Cohnella* sp., *Paenibacillus* sp., and *Pantoea* sp.	*Centella asiatica*	[44]
	*Phyllobacterium myrsinacearum*	*Epimedium brevicornu*	[45]
	*Stenotrophomonas maltophilia*, and *Bacillus* sp.	*Panax ginseng*	[46]
Improved plant abiotic stress tolerance	*Achromobacter xylosoxidans*	*Catharanthus roseus*	[47]
	*Agrobacterium* spp., and *Bacillus* spp.	*Pteris vittata*	[48]
	*Citrobacter putida*	*Euphorbia milii*	[49]
	*Glutamicibacter halophytocola*	*Limonium sinense*	[50]
	*Paenibacillus* sp.	*Plantago asiatica* and *Tridax procumbens*	[51,52]
Plant growth promotion	*Bacillus* and *Paenibacillus* spp.	*Curcuma longa*	[53]
	*Bacillus cereus* and *Bacillus subtilis*	*Teucrium polium*	[54]
	*Bacillus siamensis*	*Coriandrum sativum*	[55]
	*Micrococcus luteus* and *Lysinibacillus fusiformis*	*Panax ginseng*	[56]
	*Paenibacillus* and *Bacillus* spp.	*Lonicera japonica*	[57]
	*Serratia marcescens*	*Achyranthes aspera*	[58]
	*Variovorax* sp.	*Lavandula dentata*	[59]
Promotion of plant metabolite accumulation	*Bacillus subtilis*	*Ligusticum chuanxiong*	[60]
	*Burkholderia* sp. and *Paenibacillus polymyxa*	*Panax ginseng*	[61,62]
	*Pseudomonas fluorescens*	*Atractylodes lancea* and *Atractylodes macrocephala*	[63,64]
	*Pseudonocardia* sp.	*Artemisia annua*	[65]

**Table 2:** Summary of some techniques and criteria used to study bacterial endophytes.

Techniques	Criteria
Culture-dependent	Culture-based techniques (by plating) Advantages a. Low cost for easy microbial isolation using nutrient-rich media under specific growth conditions, allowing recovery of pure cultures effectively; b. Morphological and biochemical characterization of microbes easily achieved; c. Microbial genetic materials (DNA/RNA) easy to extract for further analysis. Disadvantages a. Laborious and time-consuming processes; b. Difficulty in assessing diverse microbial communities due to varied and specific growth requirements; c. Undesired microbial proliferation.
Culture-independent	Microscope-based techniques (TEM and SEM) Advantages a. Detailed microbial architecture, diversity, structures and colonization patterns are easily visualized. Disadvantages a. Always bulky and large in size; b. Expensive and limited to visualization under light microscope only.
	Molecular and omics-based techniques (PCR and sequencing) Advantages a. Microbial genetic materials (DNA/RNA) are easily extracted; b. Enables profiling of the complete microbiome present in the samples; c. Determines functional roles of microbes in different biological processes; d. Gives comprehensive details on the microbial functions, metabolite production, metabolic pathways, and taxonomy profiling. Disadvantages a. Plant DNA contamination during endophytic DNA extraction prior to PCR; b. Primer design and sequence analysis require prior knowledge of sequence information and skilled professionals; c. Expensive cost of primers, DNA extraction kits, and genomic sequencing; d. Low yield of endophytic DNA following 16S rRNA extraction and amplification; e. Library preparation may compromise DNA integrity, leading to errors and false results.

**Table 3:** Documented studies of omics tools and databases for bacterial endophyte analysis.

Data Platform	Type	Omics Approach	Function	Reference
QIIME2	Tool	Metagenomics	Analysis of amplicon sequencing data for microbial community profiling	[215]
MG-RAST	Tool	Metagenomics, Metatranscriptomics	Automated annotation and analysis of metagenomic and metatranscriptomic data	[216]
PICRUSt	Tool	Functional inference	Predicts functional potential of microbial communities from 16S rRNA data	[217]
KEGG	Database	All (Genomics, Transcriptomics, Proteomics, Metabolomics)	Maps genes, proteins, and metabolites to known metabolic pathways	[218]
UniProt	Database	Proteomics	Repository of protein sequences and functional annotation	[219]
GNPS	Database	Metabolomics	Annotates and visualizes microbial secondary metabolites; excellent for environmental and microbial metabolomics	[220]
MetaCyc	Database	Metabolomics	Curated database of microbial metabolic pathways and enzymes	[221]
SILVA	Database	Metagenomics	Curated rRNA gene sequence database for taxonomic classification	[222]
Greengenes	Database	Metagenomics	16S rRNA gene sequence database for bacterial taxonomy	[223]
RDP	Database	Metagenomics	Ribosomal Database Project for rRNA gene taxonomy and analysis	[224]
IMG/M	Tool and Database	Metagenomics	Integrated microbial genome/metagenome comparative analysis system	[225]

## Data Availability

All data are contained within the manuscript.

## Abbreviations

The following abbreviations are used in this manuscript:

ABA	abscisic acid
ACC	1-aminocyclopropane-1-carboxylate
AgNPs	silver nanoparticles
BLAST	basic local alignment search tool
CE-MS	capillary electrophoresis-mass spectrometry
DNA	deoxyribonucleic acid
GC-MS	gas chromatography-mass spectrometry
GNPS	global natural products social molecular networking
IAA	indole-3-acetic acid
IMG/M	integrated microbial genomes and microbiomes
ISR	induce systemic resistance
JA	jasmonic acid
KEGG	Kyoto encyclopedia of genes and genomes
LB	Luria–Bertani
LC-MS	liquid chromatography–mass spectrometry
MG-RAST	metagenome rapid annotation using subsystems technology
mRNA	messenger RNA
miRNAs	micro-RNAs
NCBI	the national center for biotechnology information
NMR	nuclear magnetic resonance spectroscopy
NPs	nanoparticles
OQDS	olive quick decline syndrome
PCR	polymerase chain reaction
PGPB	plant growth-promoting bacteria
PICRUSt	ribosomal database project
PRISMA	preferred reporting items for systematic reviews and meta-analyses
QIIME	quantitative insights into microbial ecology
RT-PCR	reverse transcription polymerase chain reaction
RNA	ribonucleic acid
rRNA	ribosomal RNA
RDP	ribosomal database project
tRNA	transfer RNA
SEM	scanning electron microscopy
TEM	transmission electron microscopy
UniProt	universal protein resource
